# Ovariectomy and overeating palatable, energy-dense food increase subcutaneous adipose tissue more than intra-abdominal adipose tissue in rats

**DOI:** 10.1186/2042-6410-2-6

**Published:** 2011-05-11

**Authors:** Viktoria Gloy, Wolfgang Langhans, Jacquelien JG Hillebrand, Nori Geary, Lori Asarian

**Affiliations:** 1 Physiology and Behaviour Laboratory, Institute of Food, Nutrition and Health, ETH Zurich, Switzerland; 2 Clinical Chemistry, Ziekenhuisgroep Twente Hengelo, the Netherlands; 3 Institute of Veterinary Physiology, Vetsuisse Faculty, University of Zurich, Switzerland

## Abstract

**Background:**

Menopause is associated with increased adiposity, especially increased deposition of intra-abdominal (IA) adipose tissue (AT). This differs from common or 'dietary' obesity, i.e., obesity apparently due to environmentally stimulated overeating, in which IAAT and subcutaneous (S) AT increase in similar proportions. The effect of menopause on adiposity is thought to be due to the decreased secretion of ovarian estrogens. Ovariectomy in rats and other animals is a commonly used model of menopause. It is well known that ovariectomy increases adiposity and that this can be reversed by estradiol treatment, but whether ovariectomy selectively increases IAAT has not been measured directly. Therefore, we used micro-computed tomography (microCT) to investigate this question in both chow-fed and dietary-obese rats.

**Methods:**

Ovariectomized, ovariectomized and estradiol treated, and sham-operated (intact) rats were fed chow or chow plus Ensure (Abbott Nutrition; n = 7/group). Total (T) AT, IAAT and SAT were measured periodically by microCT. Regional distribution of AT was expressed as IAAT as a percentage of TAT (%IAAT). Excesses in these measures were calculated with respect to chow-fed intact rats to control for normal maturational changes. Chemical analysis of fat was done in chow-fed intact and ovariectomized rats at study end. Data were analyzed by t-tests and planned comparisons.

**Results:**

Body mass, TAT, total fat mass, fat-free body mass, and %IAAT all increased in chow-fed intact rats during the 41 d study. In chow-fed rats, ovariectomy increased excess body mass, TAT, fat mass, fat-free body mass, and SAT, but had little effect on IAAT, in chow-fed rats, leading to a decrease in %IAAT. Ensure feeding markedly increased SAT, IAAT and TAT and did not significantly affect %IAAT. Ovariectomy had similar effects in Ensure-fed rats as in chow-fed rats, although less statistically reliable. Estradiol treatment prevented all the effects of ovariectomy.

**Conclusions:**

Both ovariectomy in rats and menopause are associated with increased TAT. After ovariectomy, fat is preferentially deposited as SAT and lean body mass increases, whereas after menopause fat is preferentially deposited as IAAT and lean body mass decreases. These opposite effects of ovariectomy and menopause on regional AT distribution and lean body mass indicate that ovariectomy in rats is not a homologous model of menopause-associated changes in body composition that should be used with great caution in investigations of adiposity-related diseases.

## Background

Menopause increases the risks of a number of diseases [1-3]. The abrupt and marked decrease in ovarian secretion of estrogens that occurs around menopause [4] is thought to be the main cause of these increases in health risks. In many cases, such as osteoporosis and stroke, increased risk appears to result from losses of direct estrogenic actions on the target tissues [2,5]. In others, such as type 2 diabetes mellitus and cardiovascular disease, however, decreased estrogen production also appears to increase risk indirectly, by increasing adiposity [2,6-9].

Body mass index (BMI, mass in kg/height in m^2^), axial computed tomography (CT) or magnetic-resonance imaging (MRI) scans limited to one or a few levels are commonly used measures of adiposity. A growing literature, however, indicates that these measures are not sufficiently precise to detect physiologically significant changes in the amount and regional distribution of adipose tissue (AT) [10-16]. The best available estimates of the effect of menopause on total adiposity come from studies of whole-body imaging or dual-energy x-ray absorptiometry (DEXA) scans. We are aware of six cross-sectional studies of this type in which multiple-regression analysis was used to isolate effects of menopause from those of aging *per se *[17-22]. In these studies, menopause increased body fat ~5-10% body mass. In terms of physical health, this is a substantial gain, as epidemiological data indicate that in moderately obese women, the risk of diabetes decreases 16% for each kilogram, or ~2% body mass, lost [23].

The effect of menopause on adiposity in the studies above did not appear to depend on premenopausal body mass. This is interesting because it suggests that menopause adds to other causes of increased adiposity, in particular to obesity related to the increased availability and consumption of palatable, high energy-dense foods that is thought to be the main impetus for the obesity epidemic (i.e., "dietary obesity") [24-27].

Obesity-related health risks depend on the site, as well as the amount, of AT deposition. Intra-abdominal AT (IAAT) is thought to be the most deleterious form of adiposity, and lower-body (or gluteo-femoral) subcutaneous AT (SAT), the least deleterious [28-31]. Many epidemiological studies indicate that increased waist circumference or increased waist to hip-circumference ratio is associated with increased disease risk [9,29,30,32]. Waist circumference, however, does not distinguish abdominal SAT from IAAT. This distinction requires direct measurements with CT, MRI or other imaging techniques. The effect of menopause on IAAT has been measured with whole-body imaging and analyzed by multiple regression only once. In this study, menopause increased IAAT by ~2 kg, from 4.3% of TAT in premenopausal women to 8.8% in postmenopausal women. In addition, this increase in IAAT was associated with increased signs of cardio-metabolic health risk, including fasting concentrations of plasma insulin, triglycerides and the inflammation mediators C-reactive protein and tissue plasminogen-activator antigen [21]. This is consistent with several other menopause studies using less direct measures of IAAT [3,33].

The most common model for studying the physiology of menopause is ovariectomy, which has long been known to increase body mass and adiposity in rats and mice. In several studies in which whole-body fat content was analyzed by chemical carcass analysis 4-8 wk after ovariectomy in chow-fed rats, body masses increased by means of ~35-60 g and body fat contents increased ~6-20 g [34-40]. Similar effects have been reported in mice [41,42]. The effect of ovariectomy on regional AT deposition, however, has not been clearly established in either rats or mice. Several groups have described increases in the mass of one or a few resected AT depots following ovariectomy [43-47], but none has described the total of all depots. Others have reported the total fat content of different body areas, but not of the AT depots *per se*. For example, Ainslie et al. [48], using DEXA, reported that ovariectomy increased "abdominal" and "peripheral" fat gain ~11 g each The landmarks distinguishing abdominal and peripheral, however, were not given, and, as noted, abdominal DEXA does not distinguish abdominal SAT from IAAT. Clegg et al. [36] estimated that ovariectomy increased IAAT more than SAT, but did not measure AT depots directly; rather, they resected SAT together with the skin and then estimated IAAT as the fat contents of the remaining carcass as measured by chemical analysis. In view of the fragmentary data on the effects of ovariectomy on AT mass and distribution in rats, our goal here was to provide an improved platform for the use of ovariectomy as a rodent model of menopause in obesity research. We used a combination of microCT and chemical carcass analysis to provide the first direct measures of the effects of ovariectomy on TAT, IAAT, SAT and fat outside the TAT (non-TAT fat) in chow-fed and, except for the chemical analysis, dietary-obese rats. We hypothesized that ovariectomy would affect adiposity in rats similarly to the effect of menopause in women; that is, by increasing TAT and the relative deposition of IAAT.

## Methods

### Animals

Female Long-Evans rats (bred from founders from Charles River, Sulzfeld, Germany) were housed individually in cages with wood-chip bedding, in a colony room with a 12:12 h light-dark cycle (lights off 1700 h) and an ambient temperature of 20 - 22°C. Water and ground chow were available ad libitum, except as indicated. At study onset (d 0) animals weighed 200 - 270 g and were ~12 weeks old. All procedures were approved by the Veterinary Office of the Canton Zurich.

### Measurement of AT mass

SAT, IAAT and TAT masses were measured periodically by microCT (LCT 100, Aloka, Tokyo, Japan). Rats were anesthetized with isoflurane and placed supine in the machine, and serial 2 mm scans were done from the anterior aspect of lumbar vertebra 1 to the posterior aspect of lumbar vertebra 6 (L1-6). Aloka software estimated the volumes of AT, bone, air and the remainder on the basis of their different x-ray densities, and distinguished SAT and IAAT by detecting the abdominal muscle layers. These data were converted to masses and extrapolated to whole-body SAT, IAAT and TAT masses as previously described and validated for male rats [49,50]. Whole-body scans of 44 female rats weighing 270 - 412 g were done to generate regression formulae to extrapolate L1-6 AT masses to whole-body AT masses in females. The formulae were (data in g): whole-body IAAT mass = 1.1 (L1-6 AT mass) + 1.5, (r^2 ^= 0.99); whole-body SAT mass = 3.0 (L1-6 AT mass) + 7.1 (r^2 ^= 0.91); and TAT mass = SAT mass + IAAT mass. Data below are transformed to whole-body values. Rats were assigned to one of 6 groups (n = 7 each), roughly matched for body and TAT masses on the basis of d 0 data.

### Surgery and hormone treatment

On d 1, rats were food deprived ~6 h and pretreated with 5 mg/kg trimethoprim sc and 20 mg/kg sulfadoxine sc for antibiotic prophylaxis, 50 μg/kg atropine sulfate sc, and 80 μg/kg acepromazine ip. About 20 min later, they were anesthetized with 5 mg/kg xylazine and 50 mg/kg ketamine, both ip. Four groups were ovariectomized via a 4 cm midline laparotomy and two groups were sham operated by laparotomizing them and visualizing the ovaries. Immediately after surgery, 5 mg/kg carprofen was sc injected for analgesia. This was repeated on d 2 and 3, and the antibiotic prophylaxis was repeated on d 2. Rats recovered pre-surgical body masses within 24 h. Hormone treatments began on d 5. Two groups of OVX rats received sc injections of 2 μg 17β-estradiol-3-benzoate (Sigma-Aldrich, Buchs, Switzerland; Cat # E8515) in 100 μl sesame oil (Sigma-Aldrich), and two ovariectomized groups and the two sham operated groups received oil alone. This was repeated every 4^th ^d thereafter. This estradiol regimen has been shown to elicit a near-physiological pattern of plasma estradiol concentration and to be sufficient to maintain normal food intake, spontaneous meal patterns, body mass, and (with progesterone) lordotic reflexes in ovariectomized rats [51].

### Procedure

Beginning on d 5, three groups were offered Ensure (chocolate Ensure Plus, Abbott Nutrition, Baar, Switzerland; 1.5 kcal/ml [4.7 kcal/g solids], ~28% energy from soy oil and ~57% from sugar) ad libitum in addition to chow, leading to the final allocation of rats into the six groups listed in Table 1. Ensure feeding rapidly leads to marked dietary obesity in male rats [52]. Routine maintenance, injections, body mass measurements, and vaginal cytology sampling [53] were done daily between 0900 and 1000 h. On d 0, 20, 27, 34, and 41, AT mass was measured by microCT between 1300 and 1500 h. Blood samples were mixed with EDTA, and plasma was separated and stored at -20°C. Rats were euthanized by CO_2 _inhalation on d 42. Carcasses of CH-Intact and CH-OVX rats were stored at -20°C.

**Table 1:** Group designations

Endocrine status	Diet	
	
	Chow	Chow and Ensure
Intact	CH-Intact	EN-Intact

Ovariectomized	CH-OVX	EN-OVX

Ovariectomized with estradiol treatment	CH-OVX+E2	EN-OVX+E2

### Chemical carcass analysis

Frozen carcasses of CH-Intact and CH-OVX rats were cut in 3 mm slices, lyophilized (BenchTop 2K Freeze Dryer, VirTis, Gardiner, NY, USA) to a constant mass, and homogenized in a blender. Fat content was analyzed in duplicate ~2 g aliquots by automated petroleum ether extraction (Soxtec Avanti 2050, Foss Tecator, Hamburg, Germany). In addition, in order to determine the amount of fat in rat AT, samples of inguinal, epididymal, mesenteric, omental and retroperitoneal AT from 3 male Long Evans rats were resected, combined and subjected to chemical analysis in the same run as the female samples. The CVs (mean ± SEM) of rat and AT analyses were 0.06 ± 0.05 and 0.05 ± 0.05, respectively. The recoveries of ~0.25 - 0.5 g sesame oil (Sigma) added to rat (n = 8) and AT (n = 2) samples were 0.96 ± 0.02% and 0.98 ± 0.03%, respectively, and data were corrected for this. We also compared the chemical analysis data to calculations of fat content from carcass water content, as described by Cox et al. [54], i.e., percent fat = -1.28 * percent carcass water + 95.22. The two methods agreed well: chemical extraction (g) = 0.92 * (Cox method) - 0.5, r^2 ^= 0.92, SEE = 4 g, F (1, 11) = 131.4, P < 0.05).

### Data analysis

Our design depended upon regular ovarian cycling in the intact rats and hyperphagia and the development of dietary obesity in the Ensure-fed rats. All intact rats displayed regular 4 or 5 d cycles and were included in the analysis. One Ensure-fed rat gained substantially less body mass (63 g) and TAT (21 g) than all the other Ensure-fed rats (mass gain range, 95 - 244 g; TAT gain range (46 - 107 g); body mass and TAT gains were statistical outliers (z-scores 2.34 and 2.14, P < 0.01 and < 0.016, respectively) as determined by the median-absolute deviate method as described previously [50] and the rat was excluded from the analysis. Data were analyzed only on the days of CT scans. In addition to analyzing and presenting the raw data, we also expressed them as excesses and present them in this form. This was done to exploit our longitudinal design, to increase statistical power, and to make the data more comparable to the forms recommended for studies of human obesity [55]. Excess is the difference between the changes in test groups minus the mean change in the CH-Intact control group. Subtracting the change in the CH-Intact control rats is necessary because chow-fed rats normally gain appreciable amounts of body mass and AT throughout adulthood; i.e., they are a dynamically changing control [56]. To characterize this normal maturation, the d 0 - d 41 differences in CH-Intact rats were analyzed with t-tests. Because our focus in the main analysis was on a small number of comparisons that included complex comparisons, statistical power was maximized by analyzing the data with planned comparisons. ANOVA was done to generate an experiment-wide residual error, which was used to compute standard errors of the difference (SED) and t-tests, the significances of which were determined using the Hochberg variant of the Bonferroni-Holm method [57], with an experiment-wide two-tailed α-level of P < 0.05. Five comparisons were tested: CH-OVX vs. CH-Intact, CH-OVX vs. CH-OVX+E2, EN-Intact vs. CH-Intact, EN-OVX vs. EN-Intact, and EN-OVX vs. EN-OVX+E2. Analysis of pilot data indicated that with five comparisons, we would detect as significant differences of ~10 g TAT, which we consider biologically meaningful. The variability of data from the Ensure-fed groups increased relatively faster than those of chow-fed groups, necessitating square-root or logarithmic transformation to achieve homogeneity of variance. Chemical analysis data were analyzed by t-tests. Data are reported as means ± standard errors of the mean (SEM), and SED are given to indicate experiment-wide residual errors.

## Results

### Effects of maturation and Ensure feeding

Body composition changed significantly in CH-Intact rats during the 41 d study. Body mass increased 73 ± 6 g (SED = 11 g, P < 0.001) and TAT, measured by microCT, increased 15 ± 3 g (SED = 4 g, P < 0.01) (Table 2, top row). SAT and IAAT increased in similar amounts (SAT, 7 ± 2 g, SED = 3 g, P < 0.03; IAAT, 8 ± 1 g, SED = 2 g, P < 0.01) (Table 3, top row), leading to an increase in IAAT as a percentage TAT from 41 ± 1% to 46 ± 1% IAAT (SED = 2%, P < 0.01) (Table 3). These dynamic longitudinal changes in CH-Intact rats were used to calculate changes in other groups in terms of excesses, as described in the **Data Analysis **section.

**Table 2:** Body mass and TAT mass at study onset and end

	BM (g)		TAT (g)	
	
	d 0	d 41	d 0	d 41
**CH-Intact**	226 ± 6	298 ± 9^###^	18 ± 1	33 ± 4^##^

**CH-OVX**	226 ± 6	357 ± 10*^+^	19 ± 1	51 ± 2*^+^

**CH-OVX+E2**	226 ± 7	281 ± 7	20 ± 1	34 ± 3

**EN-Intact**	226 ± 7	363 ± 7*	19 ± 1	84 ± 4*

**EN-OVX**	229 ± 7	399 ± 19^+ϕ^	18 ± 2	95 ± 10

**EN-OVX+E2**	233 ± 8	356 ± 14	20 ± 1	83 ± 7

Initial (d 0) and final (d 41) body mass (BM) and total adipose tissue (TAT) mass, determined with microCT, of chow- and Ensure-fed intact rats (CH-Intact and EN-Intact), ovariectomized rats (CH-OVX and EN-OVX), and estradiol-treated ovariectomized rats (CH-OVX+E2 and EN-OVX+E2), means ± SEM. Initial and final levels in CH-Intact rats were compared with t-tests to characterize normal maturational changes. Group differences on d 41 were analyzed by planned comparisons, with experiment-wide significance P < 0.05, as described in the text.

^#^Different from d 0, P < 0.05, ^##^P < 0.01, ^###^P < 0.001;

*Different from CH-Intact;

^+^OVX different from OVX+E2, same diet group;

^ϕ^EN-OVX different from EN-Intact.

**Table 3:** SAT mass and IAAT mass at study onset and end

	SAT (g)		IAAT (g)		IAAT (%)	
	**d 0**	**d 41**	**d 0**	**d 41**	**d 0**	**d 41**

**CH-Intact**	11 ± 0	18 ± 3^#^	8 ± 1	15 ± 2^##^	41 ± 1	46 ± 1^##^

**CH-OVX**	12 ± 0	30 ± 1*	7 ± 0	20 ± 1*	38 ± 1	40 ± 1*

**CH-OVX+E2**	12 ± 0	19 ± 2	8 ± 1	16 ± 2	40 ± 1	45 ± 1

**EN-Intact**	12 ± 1	46 ± 3*	7 ± 1	38 ± 2*	38 ± 2	45 ± 1

**EN-OVX**	11 ± 1	54 ± 6	7 ± 1	41 ± 4	39 ± 4	43 ± 1

**EN-OVX+E2**	11 ± 1	44 ± 4	8 ± 1	39 ± 2	42 ± 2	47 ± 1

Initial (d 0) and final (d 41) levels (mean ± SEM) of subcutaneous adipose tissue (SAT) and intra-abdominal adipose tissue (IAAT) masses of chow- and Ensure-fed intact rats (CH-Intact and EN-Intact), ovariectomized rats (CH-OVX and EN-OVX), and estradiol-treated ovariectomized rats (CH-OVX+E2 and EN-OVX+E2). Regional adipose tissue distribution is expressed as percentage IAAT/(SAT + IAAT). Initial and final levels in CH-Intact rats were compared with t-tests to characterize normal maturational changes. Group differences on d 41 were analyzed by planned comparisons, with experiment-wide significance P < 0.05, as described in the text.

*Different from CH-Intact;

^#^Different from d 0, P < 0.05, ^##^P < 0.01.

**Figure 1:**
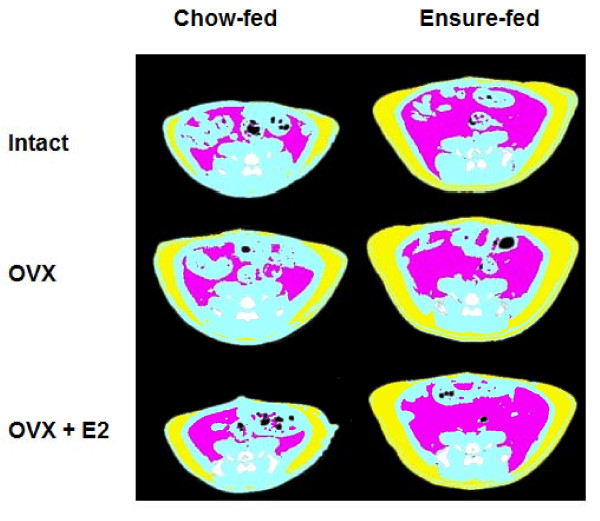
**Representative microCT images**. Representative microCT images showing SAT (*yellow*) and IAAT (*magenta*) at the level of lumbar vertebra 6 on d 41 in chow-fed and Ensure-fed rats that were sham-operated (Intact), ovariectomized (OVX), or ovariectomized and estradiol-treated (OVX+E2); *white*/*gray *is bone, *black *is air, and *blue *is the remainder.

**Figure 2:**
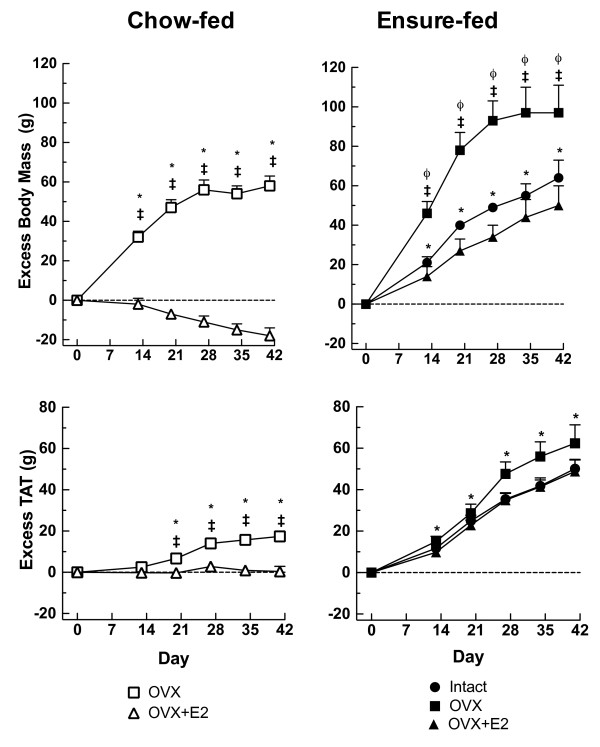
**Effects of ovariectomy and estradiol treatment on excess TAT and body mass**. Excess TAT measured by microCT and body mass were calculated with respect to the chow-fed intact group (CH-Intact, y-axis = 0), as described in the text. The left panel shows chow-fed rats that were ovariectomized (CH-OVX; open squares) or ovariectomized and estradiol-treated (CH-OVX+E2; open triangles). The right panel shows Ensure-fed rats that were sham operated (EN-intact, filled circles), ovariectomized (EN-OVX, filled squares), or ovariectomized and estradiol-treated (EN-OVX+E2, filled triangles). Data are means ± SEM and were analyzed by planned comparisons, with an experiment-wide significance of P < 0.05, as described in the text. *Different from CH-Intact; ^ǂ^OVX different from OVX-E2, same diet group; ^ϕ^EN-OVX different from EN-Intact.

**Figure 3:**
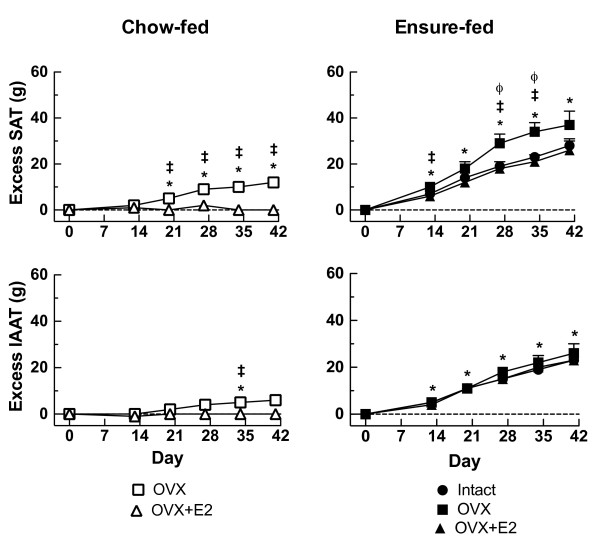
**Effects of ovariectomy and estradiol treatment on excess SAT and IAAT**. Excess SAT and excess IAAT were calculated with respect to the chow-fed intact group (CH-Intact, y-axis = 0), as described in the text. The left panel shows chow-fed rats that were either ovariectomized (CH-OVX; open squares) or ovariectomized and estradiol-treated (CH-OVX-E2; open triangles). The right panel shows Ensure-fed rats that were sham operated (EN-Intact, filled circles), ovariectomized (EN-OVX, filled squares), or ovariectomized and estradiol-treated (EN-OVX-E2, filled triangles). Data are means ± SEM and were analyzed by planned comparisons, with an experiment-wide significance of P < 0.05, as described in the text. *different from CH-Intact; ^ǂ^OVX different from OVX-E2, same diet group; ^ϕ^EN-OVX different from EN-Intact.

Ensure-feeding led to marked dietary obesity. Body mass, TAT mass, SAT mass and IAAT mass were significantly increased in EN-Intact rats compared to CH-Intact rats at study end, as shown in Tables 2 (levels of body mass and TAT) and 3 (levels of SAT and IAAT) and Figures 1 (representative microCT images), 2 (excess body mass and TAT; filled circles), and 3 (excess SAT and IAAT; filled circles). The changes were progressive: excess body mass and TAT mass were detected at each measurement point and by d 41 reached levels of 64 ± 9 g (SED = 12 g, P < 0.05) excess body mass and 50 ± 5 g (SED = 7 g, P < 0.05) excess TAT (Figure 2). Excess SAT and IAAT were increased in EN-Intact rats on each test day as well (e.g. d 41, excess SAT: 28 ± 3 g, SED = 5 g, P < 0.05; excess IAAT: 23 ± 2 g, SED = 3 g, P < 0.05) (Figure 3). IAAT as a percentage TAT on d 41 was similar in EN-Intact rats and CH-Intact rats (46 ± 1 and 45 ± 1%, respectively, SED = 2%, n.s.) (Table 3).

### Ovariectomy and estradiol treatment in chow-fed rats

Ovariectomy significantly increased body mass and TAT mass levels in CH-Intact rats (Table 2). Significant excesses in body mass were detected in CH-OVX rats on each test day and significant excesses in TAT, on d 20 and subsequently (Figure 1 representative microCT images, and Figure 2, open squares). On d 41, CH-OVX rats had 58 ± 5 g excess body mass (SED = 12 g, P < 0.05) and 17 ± 2 g excess TAT (SED = 7 g, P < 0.05). CH-OVX rats had significant excesses in SAT on d 21 and subsequently, but a significant amount of excess IAAT only on d 34 (Figure 3, open squares). As a result, IAAT as a percentage of TAT increased more slowly in CH-OVX rats than in CH-Intact rats and was significantly less on d 41 (40 ± 1% vs. 46 ± 1% in CH-OVX and CH-Intact rats, respectively, SED = 2%, P < 0.05) (Table 3). Finally, estradiol treatment significantly ameliorated all these effects of ovariectomy, except IAAT as a percentage of TAT, which was 40 ± 1% in CH-OVX rats and 45 ± 1% in CH-OVX+E2 rats (SED = 1%, n.s.), (Tables 2 and 3; Figures 2 and 3, open triangles).

Chemical carcass analysis was used to further characterize the effects of ovariectomy in chow-fed rats. At study end, CH-OVX rats had ~56 g excess body mass than CH-Intact rats (t (11) = 3.75, SED = 14 g, P < 0.01), which chemical analysis indicated was ~24 g fat (t (11) = 4.37, SED = 5 g, P < 0.01) and, by subtraction, ~33 g fat-free body mass (t (11) = 2.63, SED = 12 g, P < 0.05) (Table 4; note body mass is not identical to the values above because one rat's sample was lost from the chemical analysis). Chemical analysis of resected AT samples indicated that AT contains 84 ± 1% fat (of wet mass), which agrees well with published data [58,59]. This percentage was used to calculate the fat content of AT measured by microCT. CH-OVX rats had ~14 g more fat stored in the TAT at study end (t (11) = 3.43, SED = 4 g, P < 0.05) and ~9 g more fat stored outside the TAT (t (11) = 2.82, SED = 3 g, P < 0.05). Ovariectomy significantly changed body composition: the amounts of body fat, fat in the TAT, and fat outside the TAT normalized to fat-free body mass were all significantly increased in CH-OVX rats (t (11) = 3.68, SED = 0.018, P < 0.01; t (11) = 2.95, SED = 0.013, P < 0.05; and t (11) = 2.63, SED = 0.011, P < 0.05, respectively) (Table 4). The percentage of total body fat stored in TAT, however, did not differ significantly between CH-OVX and CH-Intact rats (t (11) = 1.34, SED = 4%, P = 0.21).

**Table 4:** Body composition data at study end

	BM	FFBM	Body fat		TAT fat		non-TAT fat	
	
	g	g	g	/FFBM	g	/FFBM	g	/FFBM
**Ch-Intact**	301 ± 11	266 ± 7	37 ± 4	0.14	28 ± 4	0.10	9 ± 1	0.03

**Ch-OVX**	357 ± 10**	296 ± 9*	61 ± 3*	0.21**	42 ± 2**	0.14**	18 ± 3*	0.06*

Fat-free body (FFBM) and body-fat masses measured at study end (d 42) by chemical carcass analysis and partitioning of fat between the TAT and non-TAT compartments of chow-fed intact (CH-Intact; n = 6) and ovariectomized rats (CH-OVX; n = 7). FFBM is body mass - body fat. Fat content of TAT was calculated using the factor of 84% determined in the present study. Data (mean ± SEM) are absolute levels and, for fat data, are also normalized to FFBM. Data were analyzed by t-tests.

*Different from CH-Intact, P < 0.05; **P < 0.01.

### Ovariectomy and estradiol treatment in Ensure-fed rats

Ovariectomy significantly increased body mass in Ensure-fed rats, both in absolute terms (Table 2) and expressed as excesses (Figure 2). TAT mass tended to increase as well, but this was not statistically significant (Figure 2; Table 2). Ovariectomy did, however, increase excess SAT in EN-OVX rats in comparison to EN-Intact rats on d 27 and 34 (9 ± 4 g and 11 ± 4 g, respectively, SEDs = 3 g, Ps < 0.05) (Table 3, Figure 3). The difference on d 41 was similar, but not significant due to increasing variability. Ovariectomy did not increase excess IAAT in EN-OVX rats in comparison to EN-Intact rats on any day; the resulting trend for a decrease in %IAAT, however, was not significant (on d 41, 43 ± 1% in EN-OVX rats and 45 ± 1% in EN-Intact rats, SED = 2%, n.s.) (Table 3). Estradiol treatment significantly ameliorated all the effects of ovariectomy that were detected in Ensure-fed rats.

Although we did not include a planned comparison to compare the effects of ovariectomy in the two diet groups (i.e., excess in CH-OVX rats vs. the difference in excesses between EN-OVX and EN-Intact rats), exploratory t-tests suggested that ovariectomy did not differentially affect either excess body mass (d 41, 58 ± 10 vs. 36 ± 19 g, t (12) = 1.04, SED = 22 g, P = 0.32) or excess TAT (d 41, 17 ± 2 vs. 26 ± 10 g, t (12) = 0.63, SED = 10 g, P = 0.54).

## Discussion

It has been known for nearly a century that ovariectomy increases adiposity in rats [60], and in recent years ovariectomy has become the most prevalent model of the increase in adiposity precipitated by menopause [61-64]. Nevertheless, many aspects of ovariectomy-induced obesity have not been well characterized. Therefore, we used microCT to determine directly for the first time the effects of ovariectomy on the development of excess AT and on regional AT distribution in chow-fed and dietary-obese rats. In addition, we further characterized the effects of ovariectomy effects on adiposity in chow-fed rats with chemical analysis of body fat and tested whether a physiological regimen of estradiol treatment was sufficient to prevent the effects of ovariectomy.

Ovariectomy produced ~56 g excess body mass in ~6 wks in the chow-fed rats that were used for chemical analysis. This consisted of ~33 g fat-free body mass and ~24 g fat. These data are similar to several previous reports [35-38], although our effects are larger than most, presumably due to the slightly longer study duration. Combining these data with the microCT data indicated that ovariectomy led to ~17 g excess TAT, of which ~14 g was fat and, consequently, ~9 g fat was deposited outside the AT. We know of only a single other report of the relative amounts of fat inside and outside the AT in rats: Tang et al. [56] found a similar effect in lean and dietary-obese male rats, although they did not emphasize this aspect of the data. Ovariectomy increased relative adiposity as well as absolute adiposity: normalized to the increase in fat-free body mass, body fat, fat in the AT, and fat outside the AT all increased significantly. The increases in absolute and relative adiposity in rats appear to parallel studies in normal-weight women revealing that menopause increases absolute and relative adiposity independent of aging [17,18,20-22].

Use of microCT also enabled us to provide the first direct measures of regional AT deposition in ovariectomized rats. Based on the limited menopause data available [21], we hypothesized that ovariectomy would increase IAAT relatively more than SAT. This hypothesis was clearly disconfirmed. In chow-fed rats, ovariectomy led to significant excess SAT (~12 g on d 41), but only non-significant excess IAAT (~6 g). This resulted in a significant reduction in the percentage of TAT deposited as IAAT compared to CH-Intact rats (~40 vs. 46%). Similarly, ovariectomy increased excess SAT clearly more than it did excess IAAT in Ensure-fed rats. We previously demonstrated that our microCT technique provides valid and accurate estimates of SAT and IAAT in rats [49]. Therefore, we conclude that ovariectomy leads to the deposition of more SAT than IAAT and, depending on the diet, may not be associated with any significant increases in IAAT. This is different from previous reports in rats that were based on less complete or indirect measurements, e.g., resection of one or a few AT depots [43-47] or DEXA of the abdominal regions [48], which does not distinguish abdominal SAT from IAAT. Clegg et al. [36] resected SAT and assumed that chemical analysis of the remaining carcass reflected IAAT, i.e., that there is little fat outside the AT. Our data indicate that this is not the case; i.e., we found that ovariectomy increased the fat content of the IAAT ~5 g and the fat content outside IAAT ~10 g. We believe that this, perhaps together with Clegg et al.'s rather small total ovariectomy effect (increases of ~32 g body mass and only ~7 g body fat over 4 wk, vs. our increases of ~54 and ~24 g, respectively), accounts for the apparent difference in results.

The selective effect of ovariectomy on SAT appears different from the effect of menopause on regional adipose tissue deposition, although the database is surprisingly thin. There has been only one whole-body imaging study with a statistical age control [21]. In this study, menopause increased IAAT about twice as much as it increased SAT. Several more limited imaging studies have reported similar results [22,33,65,66], although others have not found any selective increase in IAAT [67,68]. Given that IAAT, especially truly visceral IAAT, i.e., IAAT that drains into the hepatic-portal vein, poses the more serious challenge to metabolic health [28-30], these data suggest that the metabolic and cardiovascular consequences of ovariectomy in rats may differ importantly from those produced by menopause-induced obesity in women.

We also found that fat-free body mass increased ~33 g in chow-fed ovariectomized rats. We assume that a substantial percent of this increase represent lean body mass, as suggested by several previous reports in which lean body mass was measured directly by chemical analysis [37-40,69]. This effect is unlike menopause, which is associated with a decrease in lean body mass [17,19,20]. The metabolic milieu associated with the marked increases in lean body mass in rats vs. a loss of lean body mass in women is likely to affect many of the same metabolic variables that menopause-induced adiposity does. This seems an important issue to consider in using ovariectomy as a model of menopause, although we are unaware of studies that have done so. These different effects of ovariectomy and menopause on lean body mass may be due to differences in the effects of estrogens on growth hormone and insulin-like growth factor I. This is because ovariectomy increases and estradiol decreases secretion of both hormones in rats [70,71], whereas menopause decreases and estrogen treatment increases secretion of both [72-74]. It is not clear whether these are species differences or are related to the difference in relative age i.e., the ovariectomy data are from young adult rats, whereas menopause occurs in middle age.

We included groups of Ensure-fed, "dietary-obese" rats as a model of idiopathic human obesity, which is attributed in large part to overconsumption of palatable, high-fat, high-sugar, energy-dense food [24-27]. As expected, feeding intact rats Ensure led to further increases in excess body mass (~65 g on d 41) and TAT (~50 g). Ovariectomy led to further increases in body mass (~35 g) and TAT mass (~12 g, which was not significant). The smaller relative difference between body mass and TAT mass produced by Ensure feeding compared to the effects of ovariectomy in both chow- and Ensure-fed rats suggests that the chronic positive energy balances associated with ovariectomy and with dietary obesity were partitioned into fat and lean tissue in qualitatively different fashions. We assume that dietary obesity more closely mimics human obesity, in which there is little or no gain of lean body mass.

The effects of Ensure feeding that we observed may be to a certain extend diet-specific. This is because Lemieux et al. [37] reported that ovariectomy produced much larger effects on body mass and adiposity in rats fed a 45% sucrose, 10% fat diet than in chow-fed rats, whereas here the effects of ovariectomy were similar or smaller in Ensure-fed rats than chow-fed rats. It is important to note that both we and Lemieux et al. [37] began the dietary-obesity regimen only after ovariectomy, whereas women are more often obese before menopause. A better model of dietary obesity and menopause may be afforded by designs like that used by Noel and Fleming [75], who made rats obese by force feeding prior to ovariectomy. In this situation, ovariectomy produced similar amounts of excess body mass, which was the only obesity measure, in obese and control rats (~40 and 45 g, respectively, at d 30).

Estradiol treatment significantly ameliorated the effects of ovariectomy on the SAT, IAAT, TAT, and body mass in both diet groups. The effects on total adiposity and body mass are consistent with many previous studies [64], and the effects on SAT and IAAT are novel. These results indicate that loss of estrogen secretion is the crucial ovariectomy-induced lesion disrupting normal energy homeostasis and causing increased adiposity. That estradiol appeared to reduce body mass below intact levels was unexpected here, because this was not the case in several previous studies using the same, near-physiological estradiol regimen [36,51,76]. We have no explanation for this apparent discrepancy.

The effects of estradiol treatment on adiposity that we observed appear to parallel the effects of hormone replacement therapy (HRT) in postmenopausal women. A meta-analysis [77] of four studies [78-81] in which a total of 129 postmenopausal women were randomly allocated to HRT or to placebo or no treatment revealed that HRT decreased abdominal body fat mass by ~7% and increased lean body mass ~3%. In regard to abdominal SAT and IAAT, however, both positive [66,81] and negative [68,82] effects of HRT on IAAT have been reported in studies in which part of the abdomen was imaged. Furthermore, in two randomized trials [82,83] that not included in the meta-analysis above, together involving 128 women, no effects of HRT on fat or fat-free mass were detected. The different outcomes of these randomized trials [78-83] may be related in part to the form of HRT used. That is, HRT regimens involving larger amounts of estrogens or smaller amounts of progestins [80,81] tended to produce the larger effects. This is consistent with rat studies, which indicate that the effects of estradiol treatment on body mass in ovariectomized rats is dose-dependent and can be reduced by pharmacological progestin treatment [84].

## Conclusions

Ovariectomy in rodents is a convenient model that mimics the rapid decrease in plasma estrogens and the increase in TAT associated with menopause. Rodent ovariectomy, however, differs from menopause in two ways that are likely to produce important differences in metabolism and obesity-related pathophysiology. First, as we report here, ovariectomy preferentially increases SAT in rats, whereas menopause preferentially increases IAAT, which is metabolically more deleterious. Second, as our data suggest and others [37-40,69] have documented, ovariectomy induces gain of lean body mass, at least in relatively young rats, whereas menopause induces loss of lean body mass. These two opposite effects indicate that rodent ovariectomy is a not a homologous model of menopause-related changes in adiposity. Therefore, ovariectomy should be used with great caution in investigations of adiposity-related disease.

## List of abbreviations

AT: adipose tissue; BMI: body mass index (body mass in kg/height in m^2^); computed tomography; DEXA: dual-energy x-ray absorptiometry; EN: Ensure Plus (Abbott Nutrition); E2: 17β-estradiol-3-benzoate; HRT: hormone-replacement therapy; IAAT: intra-abdominal adipose tissue; MRI: magnetic-resonance imaging; n.s.: not significant; OVX: ovariectomy; SAT: subcutaneous adipose tissue; sc: subcutaneous; SED: standard error of the difference; SEM: standard error of the mean; TAT: total adipose tissue.

## Competing interests

The authors declare that they have no competing interests.

## Authors' contributions

VG helped in the surgeries, conducted the research, performed the CT and statistical analyses, and drafted the manuscript; WL critically revised the manuscript; JH participated in the research, helped with the CT analyses, and critically revised the manuscript; NG designed the study, helped with the surgeries, and helped draft the manuscript; LA helped design the study, performed the surgeries, participated in the research, and helped draft the manuscript. All authors read and approved the final manuscript.
